# Optimal time for the addition of non-corticosteroid immunosuppressants in myasthenia gravis: a single-center retrospective study in China

**DOI:** 10.3389/fneur.2024.1474508

**Published:** 2024-11-06

**Authors:** Jiaojiao Ma, Dan Chen, Fangfang Yi, Jie Song, Sushan Luo, Huahua Zhong, Jianying Xi, Zongtai Wu, Zunbo Li, Chongbo Zhao

**Affiliations:** ^1^Department of Neurology, Xi’an Gaoxin Hospital, Xi’an, China; ^2^Department of Neurology, The First People's Hospital of Xuzhou, Xuzhou, China; ^3^Department of Neurology, Loudi Center Hospital, Clinical Medical College of Nanhua University, Loudi, China; ^4^Huashan Rare Disease Center and Department of Neurology, Huashan Hospital, Shanghai Medical College, National Center for Neurological Disorders, Fudan University, Shanghai, China; ^5^Faculty of Biology, University of Cambridge, Cambridge, United Kingdom

**Keywords:** corticosteroid immunosuppressants, non-corticosteroid immunosuppressants, myasthenia gravis, influential factor, retrospective study

## Abstract

**Introduction:**

Patients with myasthenia gravis (MG) display strong treatment heterogeneity. Recent studies have indicated that low-dose steroids or immunosuppressants are effective. However, factors affecting the add-on of non-corticosteroid immunosuppressants to corticosteroids remain unknown.

**Method:**

Consecutive patients with MG were retrospectively reviewed from May 15, 2015, to December 29, 2020. We included one group of patients with steroid treatment alone and another group who transitioned to non-steroid immunosuppressant therapy. Clinical features of the included patients were analyzed. Univariate and multivariate Cox regression models were used to identify potential influential factors.

**Results:**

A total of 107 patients with MG were analyzed, including 66 receiving corticosteroid treatment alone and 41 who subsequently also received non-corticosteroid immunosuppressant therapy. Eight potential factors were primarily selected in univariate analysis (Ps < 0.1). Achieving minimal symptom expression (MSE) within 6 months (HR: 4.424, 95%CI: 2.102–11.865), body mass index (BMI) (HR: 0.385, 95% CI: 0.186–0.797), quantitative MG (QMG) bulbar muscle score (HR: 1.553, 95% CI: 1.140–2.118), disease duration (HR: 0.987, 95% CI: 0.977–0.997) and relapse (HR: 2.638, 95% CI: 1.031–6.750) were finally identified as potential influencing factors.

**Discussion:**

We found multifactorial clinical factors were highly associated with the add-on of non-steroid immunosuppressants after steroid treatment in patients with MG. Achieving MSE within 6 months, BMI, QMG bulbar muscle score at baseline before steroid treatment, disease duration, and disease relapse may represent crucial influencing factors, which should be considered to improve the long-term prognosis for patients with MG in future studies and practice.

## Introduction

Myasthenia gravis (MG) is an autoimmune disease that affects the postsynaptic membrane of neuro muscular junctions, resulting in fluctuant weakness in various muscle groups such as the extraocular, limb, respiratory, and bulbar muscles (1). The incidence rate of MG is 0.3–2.8 cases per 100,000 people, which is estimated to affect 700,000 people worldwide (2). The onset of MG may occur at any age, while females are significantly earlier than males. The peak incidence of female patients is before 40 years old, while the male patients is after 50 old years (3). The primary antibodies involved in the pathogenesis of MG are directed against the nicotinic acetylcholine receptor (AChR) and anti-muscle-specific tyrosine kinase (MuSK). AChR antibodies account for approximately 85% of cases, whereas MuSK antibodies account for approximately 6% (4). In approximately 15% of patients with generalized MG and 50% of those with ocular MG, both MuSK and AChR antibodies are negative, which are referred to double serum negative MG (dSnMG) (5). In the dSnMG group, 2–27% of patients have antibodies directly targeting low-density lipoprotein receptor associated protein 4 (LRP4) (6). In general LPR4 antibody-positive accounted for 2%, and triple seronegative antibodies accounted for 5% (7).

Although there are several treatment options available for patients with MG (8–10), oral steroids remain the first-line treatment and one of the most commonly used immunosuppressants (11–13). However, 5–20% patients with MG are nonresponsive to steroids, and high doses can lead to several adverse effects (13–16). Therefore, it is crucial to appropriately add non-steroid immunosuppressants for these patients (17). Besides, MuSK antibody-positive patients (MuSK-MG) frequently present exacerbation and myasthenia crisis (18, 19), and current guideline recommend Rituximab as an early-line treatment (20). Rituximab targets CD20 on the surface of B lymphocytes (21), which is different from other non-steroid immunosuppressants. Therefore, we focus on more about the AChR-MG and dSnMG patients (5).

Currently, there is a consensus on the treatment goal for patients with MG, which emphasizes achieving minimal manifestation status (MMS) or better with no more than 5 mg of prednisone (22). To achieve this treatment goal, it is important to incorporate non-steroid immunosuppressants into the treatment plan for patients with MG. Recent German guidelines recommend the glucocorticoids monotherapy or in combination with other non-steroidal immunosuppressants as first-line treatment for patients with mild to moderate disease activity (23).

However, determining the appropriate time to add non-steroid immunosuppressants to the treatment for patients with MG is controversial. A previous study combined steroid and non-steroid immunosuppressants in the initial treatment (24). However, this approach may present some challenges. First, approximately 20% of patients experienced spontaneous remission lasting for an average of 5 years (25–27). Second, some patients are responsive to lower doses of steroid [Muscle Study (28)]. Therefore, the early initiation of non-steroid immunosuppressants may result in overtreatment.

Consequently, identifying patients who would benefit from the early add-on of nonsteroid immunosuppressants is a critical issue. Accordingly, we conducted a retrospective study to explore the predictive factors for the add-on of non-steroid immunosuppressants, with the aim to provide valuable information for clinical practice.

## Materials and methods

### Patient and data collection

We retrospectively collected the clinical and follow-up data of patients with MG who were admitted to the Department of Neurology, Huashan Hospital, from May 15, 2015 to December 29, 2020. The patients were immunotherapy-naïve at baseline visit and had a minimum follow-up period of 1 year. The diagnostic criteria for MG are that the patient presents with symptoms of fluctuating weakness and has at least one positive result on the neostigmine test, serologic and electrophysiological tests (29). Patients were accessed by MG composite (MGC) scale before and after 30 min neostigmine 0.5 mg administration. The neostigmine test was defined to be positive if there was a decrease of ≥3 points in the MGC scale score after the injection compared to before (30). Patients were defined as RNS-positive when the decrease in CAMP from the first to the fourth or fifth CAMP exceeded 10% during repetitive nerve stimulation (RNS) at 2–5 Hz (31). MuSK-MG patients were excluded due to early add-on treatment with rituximab.

We evaluated the patients using the Myasthenia Gravis Foundation of American (MGFA) (32) stage, quantitative MG score (QMG) (33), quality of life (QoL) (34) score and activities of daily living (ADL) (35) scale at the first visit. The minimal symptom expression (MSE) was defined as an ADL score of 0 or 1 after 6 months from the first visit (36). The treatment strategy for steroid administration involved an initial dose of prednisone at 20 mg once a day, which was increased to 30 mg once a day after 1 week. Gradual reduction of steroid was initiated once the disease was stable. Non-steroid immunosuppressant treatment referred to oral azathioprine or tacrolimus, and was added to the steroid treatment when neurologists considered the MG symptom was not controlled well or reducing steroids was difficult.

The relapse is defined as recurrence of MG symptoms or a substantial increase in MG medications after the patient achieving MMS or better (37). The term “worsened MG” was defined as a QMG score increase of ≥4 points or ADL score increase of ≥2 points, compared to the baseline score (32). Early onset refers to the occurrence before 50 years old, while late onset indicates after the age of 50 (7). Obesity was defined as a body mass index (BMI) ≥ 28, and overweight was defined as a BMI ≥ 24 (38).

**Figure 1:**
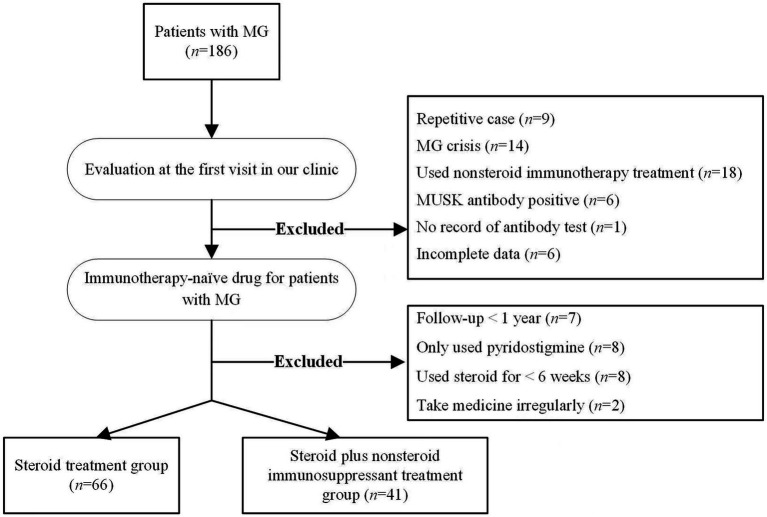
Flowchat for the selection of included patients.

The included patients were categorized into two groups on the basis of whether non-steroid immunosuppressants were added after steroid treatment: the steroid group and the combination treatment group, as shown in Figure 1.

### Serum antibody testing

Serum samples were collected from patients for antibody testing. The anti-AChR antibody titers were evaluated with an enzyme-linked immunosorbent assay (ELISA) using a commercially available kit (RSR Limited, Cardiff, UK), in which positivity was defined as at least 0.45 nmol/L. Serum MuSK antibody titers were evaluated by a commercial MuSK radioimmunoassay (RIA) antibody kit (RSR Ltd., UK). The results are expressed as nmol/L of MuSK protein bound, and positivity was defined as >0.05 nmol/L (7).

### Statistical analysis

Normally distributed measurements are described as the mean ± standard deviation (SD), while skewed data are presented as median (interquartile range, IQR). The counting data are described using frequency (percentage). The *t*-test or nonparametric test was used for the comparison of continuous measurements. Ordinal data were analyzed using a nonparametric test, while chi-square test was used for categorical data. The association between the clinical variable and outcome was analyzed using the log-rank test and univariate and multivariate Cox regression analyses. Factors with *p* < 0.1 in univariate analysis or considered clinically significant were included in the multivariate Cox regression model.

Statistical analysis was conducted using the R programming language (version 4.0.3, The R Foundation, Vienna, Austria). A two-sided *p* < 0.05 was taken to indicate statistical significance.

## Results

A total of 186 patients with MG were assessed. After applying the inclusion and exclusion criteria, 107 patients with MG were ultimately included (Figure 1). The mean onset age of MG was 38.4 ± 19.50 years, 41.12% (44/107) were males, and 58.88% (63/107) were female. Early onset of MG accounted for 78.50% (84/107), and 26.17% (28/107) had ocular MG while 73.83% (79/107) had generalized MG. Among the included patients, 93.45% (100/107) were positive for anti-AChR antibodies 6.55% (7/107) were dSnMG. During the follow-up period, 38.3% (41/107) of patients were transitioned to add-on non-steroid immunosuppressants. The transition time was 43–1,455 days after the initiation of steroid treatment, with a median transition time of 160 days. The nonsteroidal immunosuppressants used in this study included azathioprine and tacrolimus. Of the 41 patients who required add-on immunosuppressants, 46.3% (19/41) used azathioprine, 48.8% (20/41) used tacrolimus, and 4.9% (2/41) switched to tacrolimus because of adverse reactions to azathioprine.

A comparative analysis of demographic and clinical variables between the steroid group and the add-on non-steroid immunosuppressant group revealed no significant differences regarding onset age, sex, BMI, early or late onset, complicated with autoimmune disorders, onset MGFA type, MGFA type at first visit, onset symptom, AChR-ab (positive vs. negative), relapse, thymoma, thymic surgery prior to baseline visit, worsening of disease, Qol-15 score, ocular, limb QMG score at first visit, and total and each single ADL score at first visit. However, significant differences were observed in terms of disease duration, history of thymoma surgery, QMG axial muscle score, QMG bulbar muscle score, and QMG respiratory muscle score (Table 1).

**Table 1:** Baseline features of the included patients.

Variable	Corticosteroid alone (*n* = 66)	Add-on of non-corticosteroid immunosuppressant to steroid treatment (*n* = 41)	*P*
Onset age, year	39.70 ± 17.03	36.54 ± 13.86	0.320
Male, n (%)	27 (40.91)	17 (41.46)	0.955
Duration, month	35.61 ± 68.92	15.17 ± 23.85	0.030
BMI	23.22 ± 3.34	22.34 ± 3.67	0.210
Early onset, n (%) ≤ 50 y	50 (75.76)	34 (82.93)	0.380
Late onset, n (%) > 50 y	16 (24.24)	7 (17.07)	0.380
With other autoimmune diseases, n (%)	9 (13.64)	7 (17.07)	0.628
Onset MGFA type			0.556
I (%)	35 (53.03)	19 (46.34)	
II (%)	26 (39.40)	19 (46.34)	
III (%)	5 (7.57)	3 (7.32)	
MGFA type at the baseline visit			0.351
I (%)	19 (28.79)	9 (21.95)	
II (%)	28 (42.42)	18 (43.90)	
III (%)	19 (28.79)	12 (29.27)	
IV (%)	0 (0)	2 (4.88)	
Onset symptom			0.496
Ocular muscle, *n* (%)	35 (53.03)	19 (46.34)	
Limb muscle, *n* (%)	12 (18.18)	7 (17.07)	
Bulbar muscle, *n* (%)	5 (7.58)	7 (17.07)	
Generalized muscle, *n* (%)	14 (21.21)	8 (19.51)	
AChR-ab positive, *n* (%)	61 (92.42)	39 (95.12)	0.583
Relapse of disease, *n* (%)	10 (15.15)	7 (17.07)	0.792
With thymoma, *n* (%)	18 (27.27)	6 (14.63)	0.128
Thymic surgery prior to baseline visit, *n* (%)	12 (18.18)	4 (8.51)	0.235
Thymic surgery in this study, *n* (%)	19 (28.79)	5 (12.20)	0.045
Worsening of disease, *n* (%)	37 (56.05)	28 (68.29)	0.208
QOL-15 score^†^	19.92 ± 11.74	19.78 ± 11.42	0.95
QMG overall muscle score^†^	9.79 ± 4.49	11.63 ± 5.17	0.054
Ocular muscle score	3.48 ± 2.21	4.05 ± 2.29	0.209
Bulbar muscle score	0.29 ± 0.70	0.73 ± 1.25	0.017
Limb muscle score	5.05 ± 3.56	5.32 ± 3.77	0.708
Respiratory muscle score	0.11 ± 0.31	0.34 ± 0.62	0.027
Axial muscle score	0.86 ± 0.80	1.22 ± 0.76	0.025
ADL overall score^†^	5.09 ± 2.40	5.76 ± 2.42	0.167
Ocular muscle score	2.88 ± 1.84	2.98 ± 1.77	0.788
Bulbar muscle score	1.14 ± 1.49	1.63 ± 1.67	0.111
Respiratory muscle score	0.21 ± 0.41	0.22 ± 0.42	0.929
Limb muscle score	0.86 ± 1.26	0.93 ± 1.42	0.811

BMI, body mass index; MGFA, Myasthenia Gravis Foundation of America; ACHR-ab, acetylcholine receptor antibody; QOL: quality of life; QMG, quantitative myasthenia gravis; ADL, Activities of Daily Living. ^†^Baseline visit before steroid treatment.

Univariate analysis indicated that MGFA type at baseline visit before steroid administration, MSE within 6 months, BMI, QMG bulbar muscle score, QMG respiratory muscle score, QMG axial muscle score, QMG total score, and ADL bulbar muscle score were associated with the add-on of non-steroid immunosuppressant (Table 2). Multivariate analysis confirmed that BMI, MSE within 6 months, QMG bulbar muscle score, disease duration, and relapse were associated with the outcome of adding non-steroid immunosuppressants (Table 3).

**Table 2:** Univariate analysis for the add-on of non-steroid immunosuppressants to steroid treatment.

Variable	*P*	HR	95.0% CI for HR	
			Lower	Upper
Sex	0.994	0.998	0.529	1.881
Early-onset	0.249	0.599	0.251	1.430
Onset age, years	0.184	0.987	0.967	1.006
Duration	0.199	0.994	0.985	1.003
Onset MGFA type	0.394	1.235	0.760	2.006
MGFA type at baseline	0.097	1.432	0.937	2.188
MSE within 6 months	< 0.001	4.134	1.734	9.854
MSE within 1 year	0.421	1.292	0.692	2.409
With other auto-immune disease	0.686	1.006	0.979	1.033
Worsening of disease	0.398	1.331	0.686	2.581
Relapse of disease	0.502	1.323	0.584	2.997
With thymoma	0.957	1.040	0.249	4.354
Thymic surgery in this study	0.329	0.597	0.213	1.679
Thymic surgery prior to the baseline visit	0.389	0.635	0.226	1.786
AChR-ab positive	0.221	3.462	0.474	25.265
BMI	0.044	0.477	0.233	0.981
QMG ocular muscle score	0.144	1.111	0.965	1.280
QMG bulbar muscle score	0.007	1.461	1.108	1.926
QMG limb muscle score	0.505	1.031	0.942	1.128
QMG respiratory muscle score	0.006	2.054	1.227	3.438
QMG axial muscle score	0.012	1.641	1.114	2.418
QMG overall score	0.016	1.089	1.016	1.167
QOL-15 score	0.686	1.292	0.979	1.033
ADL bulbar muscle score	0.057	1.202	0.994	1.453
ADL respiratory muscle score	0.767	0.894	0.425	1.880
ADL limb muscle score	0.538	1.078	0.849	1.369
ADL ocular muscle score	0.957	1.005	0.849	1.188
ADL overall score	0.124	1.105	0.973	1.256

MGFA, Myasthenia Gravis Foundation of America; MSE, minimal symptom expression; AChR-ab, acetylcholine receptor antibody; BMI: body mass index; QMG, quantitative myasthenia gravis; QOL, quality of life; ADL, Activities of Daily Living.

**Table 3:** Multivariate analysis for the add-on of non-steroid immunosuppressants to steroid treatment.

Variable	*P*	HR	95%CI for HR	
			Lower	Upper
BMI	0.010	0.385	0.186	0.797
MSE within 6 months	0.001	4.424	2.102	11.865
QMG bulbar muscle score	0.005	1.553	1.140	2.118
Disease duration	0.010	0.987	0.977	0.997
Relapse of the disease	0.040	2.638	1.031	6.750

BMI, body mass index; MSE, minimal symptom expression; QMG, quantitative myasthenia gravis.

**Figure 2:**
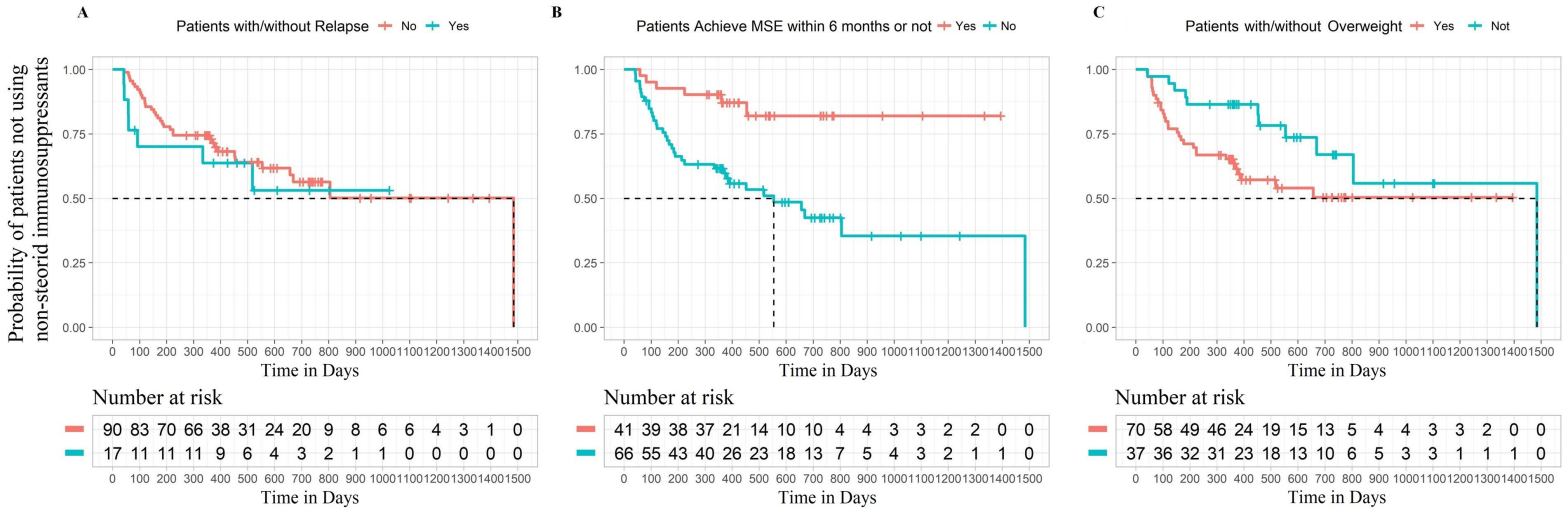
Survival curves for time of addition of non-steroid immunosuppressive treatment in patients with different characteristics (The horizontal axis represents follow-up time, and the vertical axis represents survival rate).

For categorical features, we also used the K-M plot to illustrate the differences in survival outcome between patients with different relapse statuses, BMI categories, and MSE status at 6 months (Figure 2). As shown in Figure 2A, the average time from initial steroid treatment to the add-on of non-steroid immunosuppressants was significantly shortened in patients with MG with relapse (log-rank *p* < 0.05). It was demonstrated that patients with MG achieved MSE within 6 months, which required a longer time for adding non-steroid immunosuppressants (Figure 2B, log-rank *p* < 0.05). For patients who did not achieve the MSE at 6th month, the survival curve declined much more rapidly compared to those who did. We can observe a sharp decline starting from the very beginning of the follow-up, which gradually reached a relative stable status after about 800 days. Overweight patients were more likely to add non-steroid immunosuppressant than non-overweight patients (Figure 2C, log-rank *p* < 0.05).

## Discussion

MG is a heterogeneous disease with various treatment options available (39). Despite the increasing use of immunotherapy, steroids remain the first-line treatment for MG (11, 17). A previously published study on 116 patients with MG treated with steroids found that 80.2% achieved remission or remarkably improved (13). Moreover, several studies have reported that low doses of steroids are effective in some patients with MG (Muscle study (28)). Previous studies have also revealed that symptoms of MG can be improved by a single use of steroids (11). However, it has also been reported that 5–20% of patients with MG are nonresponsive to steroid treatment (14–16).

Treatment with non-steroid immunosuppressants plays an important role in inadequate response or unresponsive patients (40). Recent studies have demonstrated that azathioprine, mycophenolate mofetil and tacrolimus are widely used and may be effective as monotherapy for patients with MG (9, 10, 41). Many studies indicated adding non-steroidal immunosuppressive agents may help reducing the dosage of steroids (37, 42). However, the decision of when and whether to add immunosuppressants remains a topic of debate. One study combinated of steroids and non-steroid immunosuppressants in the initial MG treatment (24). In this study, we explored the factors associated with the addition of non-steroid immunosuppressants to steroid therapy. Our results indicated that MSE within 6 months, BMI, QMG bulbar muscle score, disease duration, and relapse of illness are influencing factors that suggest the need for the addition of non-corticosteroid immunosuppressants to corticosteroids in patients with MG.

Our results suggested that high BMI (≥24 kg/m^2^) is a risk factor for the add-on of nonsteroidal immunosuppressants. Indeed, obesity is a known risk factor for autoimmune diseases such as asthma, rheumatoid arthritis, and psoriasis (43, 44). Obese patients have low sensitivity to steroid treatment, and weight loss might improve the response to steroid treatment (45). A cross-sectional study focusing on the association between obesity and patients with MG has indicated an increased risk of obesity in patients with MG, regardless of corticosteroid treatment (46). Based on our findings, we supposed that obese patients with MG may be less sensitive to corticosteroid therapy, which requires an early combination of non-corticosteroid immunosuppressants. Obesity may affect sensitivity to steroid treatment through various mechanisms. First, increased body fat could lead to increased levels of inflammation (47), affecting the body’s ability to respond to steroid treatment. Obesity can also change the distribution of fat (61), leading to changes in steroid absorption and metabolism, potentially affecting the effectiveness of steroid therapy. Additionally, obesity is often associated with insulin resistance, which interferes with the ability of steroids to target receptors (48), thus reducing the effectiveness of steroid treatment. However, further research is needed to better understand the association between obesity and MG.

In clinical practice, the QMG score is an important tool for evaluating disease severity and treatment response in patients with MG (49). Indeed, Li et al. (50) suggested that patients who achieved MMS may have lower baseline QMG scores than those who did not, and a low QMG score (QMG score ≤ 16 points) may be regarded as a potential predictor of reaching MMS. A study on the prediction of postoperative crisis in patients with thymoma showed that bulbar symptoms were a risk factor for developing postoperative crisis (OR = 7.24), although without controlling potential covariates (51). Su et al. (53) found that the onset of bulbar symptoms is a risk factor for disease relapse during the glucocorticoid reduction and withdrawal period for patients with MG who reached MMS. Therefore, with a high baseline QMG score, particularly for a high bulbar QMG score, it is crucial to add a non-steroid immunosuppressant to reduce relapse and achieve MMS, which corresponds to our study. Besides, the respiratory function or respiratory QMG score might be an influence factor for the need of add-on non-steroid immunosuppressant therapy. A recent study showed that respiratory involvement was more common in those taking prednisone (52). In our study, although there was no significant difference in multivariate analysis, it is necessary to add a non-steroid immunosuppressant for those with a high baseline respiratory QMG score in univariate analysis.

Recent research has indicated that ADL is highly associated with QMG scores (*r* = 0.726) (53). Previous studies have shown that 70% of patients with MG reach the most severe state of disease in the first year (13). The median time for female and male patients to achieve maximum improvement is 6 and 5 months for female and male patients, respectively (13). Therefore, we selected MSE within 6 months and MSE within 1 year as potential influencing factors, and found that patients with MSE within 6 months are less likely to receive non-steroid immunosuppressants, which might provide more evidence for the decision of whether early non-immunosuppressant treatment is needed.

Our results also suggested that disease duration before immunotherapy is associated with MG prognosis. However, there is inconsistency in the current understanding (54). Some studies have indicated that long disease duration is a risk factor for poor prognosis for postoperative crisis of thymoma (51), while others have shown that a long disease duration is associated with remission in medication therapy (54). Our results showed that a long disease duration before steroid treatment is negatively associated with the add-on of non-steroid immunosuppressants. This study may indicate that patients with a long disease duration before immunotherapy were relatively stable. Besides, ocular patients with MG, who have a disease duration of more than 2 years, currently are considered in a relatively stable status (27). Another study indicated that generalized MG exhibited a longer duration than ocular MG, along with a higher proportion of patients receiving immunosuppressant therapy (52). This implies that the subtype classification of MG could also be a significant factor influencing the need for increased immunosuppressive therapy.

Previous studies demonstrated that 18–34% of patients with MG will experience a relapse (13, 37, 55). A study has reported that patients who have a shorter duration of corticosteroid reduction, specifically less than 11.5 months, are more prone to relapse (53). It has been suggested that a proportion of patients with MG are dependent on steroid treatment. A study of nine patients with steroid-dependent MG showed that cyclosporine can improve symptoms and prevent relapse after steroid reduction or withdrawal (56), suggesting that cyclosporine can reduce steroid dependence in patients with MG. Another comparative study, which compared rapid and slow steroid reduction with the use of azathioprine, found that the rate of disease relapse within 15 months was not affected, suggesting that azathioprine can reduce the steroid dependency in patients with MG (57). These findings suggest that the add-on of non-steroid immunosuppressants to steroid treatment may be necessary for patients with MG experiencing a relapse. Furthermore, these results imply that patients with disease relapse may not be responsive to or reliant on steroid treatment.

Recently, new treatment options as potential steroid-sparing agents have garnered significant attention. Among these, FcRn inhibitors and complement inhibitors are expected to play a central role in the treatment of MG (58–60). A recent real-world experience study demonstrated that both efgartigimod and eculizumab show significant efficacy in improving patient symptoms and reducing steroid dependence, indicating a promising direction for future treatments (59).

### Limitation of the study

There are several limitations that are worth considering. First, MuSK-MG was not included, which may limit the generalizability of the findings. Second, the retrospective nature of this study, combined with a relatively small sample size, raises concerns regarding potential selection bias among the participants. Furthermore, the limited follow-up duration may have resulted in the oversight of patients who are reliant on steroids, thereby affecting the comprehensiveness of the data. These factors should be taken into account when interpreting the results of this study.

## Conclusion

In conclusion, our findings suggest that patients with MG who achieve MSE within 6 months, without experiencing relapse, and have a normal or lean weight may respond well to treatment with a monotherapy of steroid. However, for patients who do not meet these criteria, it may be necessary to consider the add-on of nonsteroidal immunosuppressant treatment.

## Data Availability

The data supporting the findings of this study can be obtained from the corresponding author upon reasonable request.
